# Physicochemical Properties and Hematocompatibility of Layered Double Hydroxide-Based Anticancer Drug Methotrexate Delivery System

**DOI:** 10.3390/pharmaceutics12121210

**Published:** 2020-12-14

**Authors:** Sang-Yong Jung, Hyoung-Mi Kim, Soonjae Hwang, Do-Gak Jeung, Ki-Jong Rhee, Jae-Min Oh

**Affiliations:** 1 Department of Energy and Materials Engineering, Dongguk University-Seoul, Seoul 04620, Jung-gu, Korea; syjung05@dgu.ac.kr (S.-Y.J.); jdk941101@naver.com (D.-G.J.); 2 Department of Chemistry and Medical Chemistry, Yonsei University MIRAE Campus, College of Science and Technology, Wonju 26493, Gangwon-do, Korea; annabb@hanmail.net; 3 Natural Product Informatics Research Center, Korea Institute of Science and Technology, Gangneung 25451, Gangwon-do, Korea; soonjae@kist.re.kr; 4 Department of Biomedical Laboratory Science, Yonsei University MIRAE Campus, College of Health Sciences, Wonju 26493, Gangwon-do, Korea

**Keywords:** colloidal property, in vivo, layered double hydroxide, hemolysis, particle size

## Abstract

A layered double hydroxide (LDH)-based anticancer delivery system was investigated in terms of crystalline phase, particle size, hydrodynamic radius, zeta potential, etc. through in vitro and in vivo study. Size controlled LDH with anticancer drug methotrexate (MTX) incorporation was successfully prepared through step-by-step hydrothermal reaction and ion-exchange reaction. The MTX-LDH was determined to have a neutral surface charge and strong agglomeration in the neutral aqueous condition due to the surface adsorbed MTX; however, the existence of proteins in the media dramatically reduced agglomeration, resulting in the hydrodynamic radius of MTX-LDH being similar to the primary particle size. The protein fluorescence quenching assay exhibited that MTX readily reduced the fluorescence of proteins, suggesting that the interaction between MTX and proteins was strong. On the other hand, MTX-LDH showed much less binding constant to proteins compared with MTX, implying that the protein interaction of MTX was effectively blocked by the LDH carrier. The in vivo hemolysis assay after intravenous injection of MTX-LDH showed neither significant reduction in red blood cell number nor membrane damage. Furthermore, the morphology of red blood cells in a mouse model did not change upon MTX-LDH injection. Scanning electron microscopy showed that the MTX-LDH particles were attached on the blood cells without serious denaturation of cellular morphology, taking advantage of the cell hitchhiking property.

## 1. Introduction

Nanotechnology has affected science and technology in many aspects during the recent decades, and pharmaceutics is one of the most strongly influenced field. Nanomedicine, which consists of nanoimaging, nanomaterials/devices, nano delivery system, therapeutics etc. [1,2,3,4,5], evoked new methodologies in pharmaceutics, suggesting unprecedented solutions to serious problems. Various nanoparticle-based delivery systems, such as self-assembled systems, supramolecular assembly, polymers, and inorganic materials, have been developed [6,7,8,9,10,11]. Among them, layered double hydroxide (LDH) is one of the fascinating inorganic nano delivery carriers due to its unique structure and potential surface functionalization. LDH is composed of positively charged inorganic metal hydroxide layers, which have ~0.5 nm thickness and tens to hundreds of nanometers in lateral size, and interlayer anions [12,13,14]. The layer-by-layer stacking between inorganic layers and interlayer anions is stabilized by electrostatic interaction, developing several tens of layers intervened by each other. It has been reported that LDH can accommodate a large amount of anionic drugs with high biological inertness and physicochemical stability [15,16,17,18]. The particle size of LDH can be easily controlled through adopting an appropriate synthesis route—coprecipitation, hydrothermal treatment, urea hydrolysis etc. [19]—to have the optimum dimension for low systemic toxicity and high cellular delivery efficacy [18,20]. Incorporated drug or gene moieties are released in a controlled manner under weakly acidic conditions to provide target cells with payloads in a sustained manner [21,22]. Furthermore, the surface of LDH can be functionalized with various groups to enhance target specificity [23,24,25].

Due to the above-mentioned advantages, there have been extensive research studies on LDH-based drug delivery systems utilizing the anticancer drug, methotrexate (MTX). The incorporation of MTX into LDH (MTX-LDH) [26] and the controlled release of MTX in an intracellular environment have been reported [22,27,28]. The MTX-LDH dramatically enhanced the anticancer effect of MTX towards various cultured cell lines such as KB, A549, Saos-2, MNNG-HOS, MG-63, Hep1 etc. [5,23,29,30,31,32], which was mainly attributed to the clathrin-mediated endocytosis [5]. The loaded drug moiety can be stably stored under neutral physiological conditions due to the protein corona formation at the LDH surface preventing drug release. Once the MTX-LDH particles enter a cancer cell through endocytosis, they are sequentially located in endosomes and lysosomes where the protein corona is detached and the LDH lattice is partially dissolved under acidic conditions. In those intracellular organs, the loaded drug moiety would be released through partial lattice dissolution and anion exchange [22,30]. The successful cellular delivery function of LDH even overcame drug resistance, which strongly prevents the repetitive drug efficacy of anticancer agents [32]. Furthermore, the MTX-LDH system has been recently applied to hybrid therapeutics such as chemo-photothermal therapy [33], combination therapy [31] etc.

Although the cytotoxic effect and cellular delivery of MTX-LDH have been reported in many pieces of literature, there still remains problems to be solved regarding the systemic efficacy, tumor specificity and colloidal properties of nanoparticles. Fortunately, size-controlled MTX-LDH particles, which are injected to mice models through the tail vein, are reported to accumulate in the tumor tissues [34,35], possibly due to the enhanced permeation and retention (EPR) effect. However, the latter one, the changeable colloidal properties of nanoparticles depending on media, is one of the concerns to be addressed, especially as the LDH-based drug delivery system is aiming at intravenous injection and thus the colloidal properties in the blood vessel environment should be investigated. Nanoparticles generally form protein corona, which facilitates its cellular uptake [36]; on the other hand, the protein adsorption onto certain kinds of nanoparticle could induce conformational change of proteins [37], resulting in potential toxicity. Nanoparticles such as fullerene derivatives or Ag-nanoparticles are known to induce membrane damage on red blood cells [38,39], while several nanoparticles including TiO_2_, Ag and nanodiamonds were reported to affect morphological change in blood cells [40,41,42,43].

There have been several reports on the colloidal properties of MTX-LDH [44]; however, the research studies were focused on the size control of MTX-LDH for homogeneous particle distribution in aqueous media. In order to address the safety and stability of MTX-LDH in blood vessel conditions, its colloidal behaviors under the existence of protein and the in vivo compatibility should be comprehended in parallel. In this study, we are going to demonstrate the zeta potential and hydrodynamic radius of size controlled MTX-LDH nanoparticles to understand the colloidal properties. Then, the interaction between MTX-LDH and proteins is quantitatively analyzed to examine its behavior in blood condition. Finally, in vivo hemolysis and morphological change of blood cells after MTX-LDH injection are investigated in an animal model.

## 2. Materials and Methods

### 2.1. Materials

Magnesium nitrate hexahydrate (Mg(NO_3_)_2_·6H_2_O), aluminum nitrate nonahydrate (Al(NO_3_)_3_·9H_2_O), sodium nitrate (NaNO_3_) and MTX were purchased from Sigma-Aldrich Co. LLC. (St. Louis, MO, USA). Sodium hydroxide pellets (NaOH) were obtained from Daejung Chemicals & Metals Co., Ltd. (Siheung, Korea). Human serum albumin (HSA, cat. no. A9511), globulin (cat. no. G4386) and fibrinogen (cat. no. F4129) were purchased from Sigma-Aldrich Co. LLC. (St. Louis, MO, USA).

### 2.2. Synthesis of Pristine LDH and MTX-LDH

First, MgAl-NO_3_-LDH was prepared by coprecipitation and hydrothermal treatment. A metal solution containing Mg(NO_3_)_2_·6H_2_O and Al(NO_3_)_3_·9H_2_O (2:1 in molar ratio) was prepared and titrated with 0.9 M NaOH containing 0.5 M NaNO_3_ until pH ~9.5 under nitrogen atmosphere. The mixture was transferred to a Teflon-lined autoclave for hydrothermal treatment. After 12 h of treatment at 473 K, the white precipitate was separated by centrifugation (6000 rpm, 5 min), thoroughly washed with decarbonated water, and stored as it was.

To synthesize MTX-LDH, powdered MTX was dispersed in decarbonated water, and titrated by NaOH (0.5 M) solution to give clear MTX solution at pH ~7. Then, the previously obtained MgAl-NO_3_-LDH filter-cake was re-suspended in decarbonated water and mixed with MTX solution keeping an MTX/Al ratio of ~1.2. Due to the concentration gradient of MTX between the outside and inside of LDH, the outer MTX gradually replaced the interlayer nitrate. After 24 h of reaction under nitrogen atmosphere, the yellow precipitate was separated by centrifugation (6000 rpm, 5 min) and thoroughly washed with decarbonated water. A part of MTX-LDH was lyophilized for physicochemical characterization and the other part was stored as it was for further biological assays.

### 2.3. Characterization of MTX-LDH

The incorporation of MTX in the gallery space of LDH was investigated by a powder X-ray diffractometer (PXRD: D2 Phaser, Bruker AXS GmbH, Karlsruhe, Germany) with Ni-filtered Cu Kα X-ray (λ = 1.5406 Å). Powdered samples (~200 mg) were packed in the glass and the upper part of the powder was mounted with flat slid glass. Diffractograms were obtained in the 2θ range 5–30° for MgAl-NO_3_-LDH and 3–20° for MTX-LDH, respectively, with time step increments of 0.02° and 0.2 s/step. In order to examine the chemical functional groups in MTX-LDH, Fourier-transform infrared spectroscopy (FT-IR) was carried out for both MgAl-NO_3_-LDH and MTX-LDH, utilizing the attenuated total reflectance (ATR) technique (Spectrum 65 FT-IR spectrometer, PerkinElmer, Massachusetts, MA, USA). A small amount (10~20 mg) of powder was located on the sample stage and gently pressed with a diamond head for measurement. The measurements were repeated 4 times under a resolution of 4 cm^−1^ in the wavenumber range 400–2000 cm^−1^. The background spectrum coming from air was automatically subtracted in the instrument.

The particle size and shape of MTX-LDH were observed by scanning electron microscopy (SEM: FEI QUANTA 250 FEG, Hillsboro, OR, USA) at 30 kV of accelerating potential. The powdered sample (~10 mg) was directly put on a sticky carbon tape, which was attached on the top of the sample stub. The powder was gently spread with a wooden toothpick and the loosely bound powders were removed by a hand air-blower. Then, the surface of sample was coated with Pt/Pd plasma sputtering for 50 s. The SEM images were obtained at a random point of the sample for the statistical analyses of particle size. The histogram of size distribution was obtained from the randomly selected 100 particles in SEM images, and the normal distribution was calculated with Microsoft Excel^®^.

The hydrodynamic radius and zeta potential of MTX-LDH were measured by Otsuka electronics ELSZ-1000 (Otsuka electronics, Osaka, Japan) in a neutral aqueous condition and simulated plasma condition. The plasma condition was prepared by dissolving human serum albumin in phosphate buffered saline (PBS). The concentration of albumin was set at ~0.4 g/mL considering the actual albumin concentration in plasma. The concentration of optimal suspension was 0.1 mg/mL for the hydrodynamic radius and zeta potential.

### 2.4. In Vitro Biological Assay: Plasma Protein Fluorescence Quenching

In order to investigate the potential interaction between samples (MTX, LDH, and MTX-LDH) and representative blood proteins (albumin, fibrinogen, and globulin), the fluorescence quenching effect was evaluated at various sample concentrations. Each protein was dissolved in calcium and magnesium free Dulbecco’s phosphate buffered saline (DPBS) at a concentration of 1 mg/mL. Samples (MTX, LDH, and MTX-LDH) were dissolved or suspended in DPBS at concentrations of 0, 0.4, 0.8, 1.2, 1.6, 2.0, 3.0 and 4.0 mg/mL. The protein solution and sample were mixed at 1:1 volume ratio and were placed on a Thermo Finemixer (FINEPCR SH2000-DX, FINEPCR, Gunpo, Korea) at 36.5 °C for 0.5 h. Then, the fluorescence intensity at 360 nm under an excitation wavelength of 280 nm was measured utilizing a luminescence spectrometer (Perkin Elmer LS55, PerkinElmer, MA, USA).

The fluorescence quenching pattern was quantitatively analyzed based on the Hill equation [45,46] as below.

Q/Q_max_ = [L]^n^/(K_D_^n^ + [L]^n^) (1)

Q: quenching ratio = (I_0_ − I)/I_0_ (I_0_ and I stand for fluorescence emission intensity without and with quencher); Q_max_: maximum quenching ratio at equilibrium; [L]: concentration of quencher, i.e., MTX, LDH or MTX-LDH in this study; K_D_: dissociation constant = 1/K_b_ (binding constant); n: Hill parameter related to cooperativity.

The above equation was converted as follows for linear regression.

log(Qmax/Q − 1) = −nlog[L] + nlogK_D_ (2)

### 2.5. In Vivo Biological Assay: Hematocompatibility in Mouse Model

The animal test was carried out under the guidance of the Institutional Animal Care and Use Committee (IACUC), Yonsei University-Wonju (YWCI-201607-003-01, approved on 11th July 2016). The MTX-LDH was suspended in saline to obtain a 10 mg/mL concentration, then injected into six respective Balb/c mice utilizing a 31 G sterilized syringe (100 μL). After 0.5 and 6 h, three mice at each time point were euthanized with CO_2_ gas. Then, blood was collected directly from the cardiac puncture utilizing a 26 G sterilized syringe (~50–500 μL) and stored in a citrate coated vial. Three mice without MTX-LDH injection were euthanized and the blood was collected as above for the control group.

Hemolysis of the mouse with or without administration of MTX-LDH was evaluated by modifying hemolysis assay of ASTM F756, as follows. The blood samples collected from the mouse after 0, 0.5 and 6 h or MTX-LDH were mixed with saline in a 1:1 volume ratio and placed at room temperature for 0.5 h to change hemoglobin to oxyhemoglobin. The samples were then centrifuged at 3000 rpm for 5 min and the absorbance of supernatant at 540 nm was recorded utilizing UV–vis spectroscope (SHIMADZU UV-1800, Shimadzu Schweiz GmbH, Reinach BL, Switzerland). The blood with deionized water instead of saline and the one collected at 0 h were utilized as positive (100% hemolysis) and negative control (0% hemolysis), respectively. The number of viable cells in the collected blood was also counted for quantitative analysis. The collected blood was diluted 200 times with saline and the number of cells in a chamber (9 mm^2^) of the hemocytometer was determined through counting, which was repeated 5 times.

In order to investigate the change in the morphology of the blood cells and the possible interaction between MTX-LDH and blood cells, both optical microscopy at low magnification (Korea lab tech, Seong-Nam, Korea) and scanning electron microscopy at high magnification (FEI QUANTA 250 FEG, Hillsboro, WA, USA) were carried out. The blood samples collected from the mouse model at each time point were located on the slide glass, smeared, fixed with methyl Wright’s stain solution (cat. no. 1503-3, Muto pure chemicals, Tokyo, Japan) for 4 min, and stained with Wright’s stain buffered at pH 6.4 for 6 min. The slides were directly subjected to optical microscopy in order to check the morphological change. A piece of slide glass was sputtered with Pt for 20 sec for SEM study (FEI QUANTA FEG250, FEG, Hillsboro, WA, USA).

## 3. Results and Discussion

**Figure 1:**
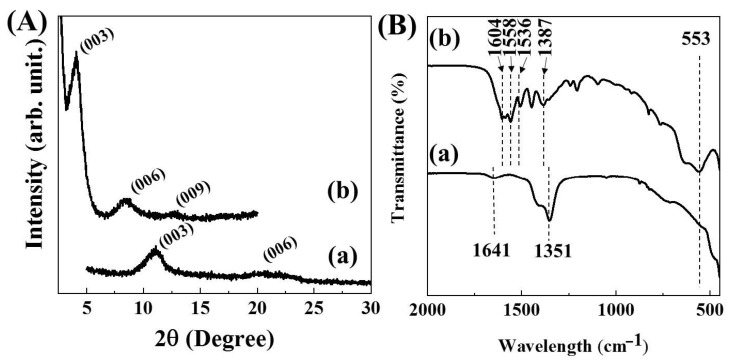
(**A**) X-ray diffraction patterns and (**B**) Fourier-transform infrared spectra for (a) MgAl-NO_3_-layered double hydroxide (LDH) and (b) methotrexate (MTX)-LDH. The XRD patterns were focused on the low angle region to highlight (00l) patterns.

The powder X-ray diffraction patterns in the low angle region clearly showed lattice expansion of LDH along the crystallographic c-axis. The diffractogram of MgAl-NO_3_-LDH exhibited (Figure 1A(a)) characteristic (00l) diffraction patterns of layered materials showing a (003) peak at 10.5° and (006) peak at 23.0°, respectively. The calculated d-spacing from Bragg’s equation was 0.84 nm, which corresponded to the d-spacing of the previously reported NO_3_-LDH [17]. On the other hand, the diffractogram of MTX-LDH showed a dramatic peak shift to the lower angle region, showing (003), (006), and (009) peaks at 4.1°, 8.4°, and 12.7°, respectively (Figure 1A(b)), suggesting the expansion of d-spacing up to 1.88 nm, which was comparable with the previous reports [26,35,44]. The encapsulation efficiency and loading capacity of MTX was ~75% and ~53 wt/wt%, respectively, suggesting that the interlayer space of LDH was effectively filled with MTX molecules.

In order to evaluate the chemical property of MTX-LDH, the FT-IR spectra were obtained for both MgAl-NO_3_-LDH and MTX-LDH. The infrared spectrum of MgAl-NO_3_-LDH exhibited a strong absorption peak at 1351 cm^−1^ and a small band at 1641 cm^−1^, attributed to the stretching vibration mode of interlayer nitrate and bending of adsorbed water, respectively (Figure 1B(a)) [47,48,49]. The characteristic band at the low energy region, the broad band at 553 cm^−1^, was attributed to the metal–oxygen vibration stretching in the framework of the LDH lattice [48]. On the other hand, the spectrum of MTX-LDH (Figure 1B(b)) exhibited characteristic absorption peaks that originated from the organic moiety in MTX. The peaks at 1604 and 1558 cm^−1^ were assigned to N–H bending and C–N stretching in MTX moiety, respectively [26,50,51], suggesting that the intact structure of MTX was well preserved after the ion-exchange reaction. Furthermore, we could observe two peaks at 1387 and 1536 cm^−1^ corresponding to the symmetric and antisymmetric stretching vibration of carboxylate [52]. The carboxylic acid groups in MTX molecules were deprotonated before the ion-exchange. The IR result exhibited that the deprotonation was maintained after incorporation into LDH in order to maximize electrostatic interaction between the positive charge of LDH and negative charge of MTX.

**Figure 2:**
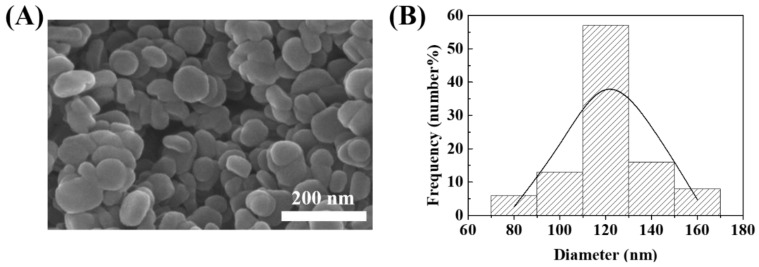
(**A**) Scanning electron microscopic (SEM) image and (**B**) corresponding particle size distribution for MTX-LDH. The SEM images were directly obtained from the powdered sample without dispersion in solvent. The acceleration voltage was set as 30 kV. The histogram of size distribution (bar with diagonal bars) was obtained from the randomly selected 100 particles in the SEM images, and the normal distribution (solid line) was fitted with normal distribution function in Microsoft Excel^®^.

As the particle size and homogeneity are important factors regarding the toxicology of nanoparticles, a SEM study was carried out for the MTX-LDH powder and the size distribution was obtained (Figure 2). It was revealed that the MTX-LDH particles have a pebble-like shape with a relatively large lateral size compared to thickness (Figure 2A), which is a typical shape of hydrothermally synthesized LDH particles [15,18,20]. Approximately 100 particles were randomly selected from the SEM images and the size distribution in terms of lateral size was illustrated with a histogram. The average particle size was 122.3 nm, and the standard deviation was 17 nm. According to the previous study, the particle size of LDH between 100–200 nm is advantageous in drug delivery systems in terms of cellular uptake [53], high therapeutic efficacy [35], low toxicity etc., in both cultured cell lines and an in vivo mouse model [54]. Therefore, the current MTX-LDH was considered to be the optimized sample to test the hematocompatibility of the LDH-based drug delivery system. In order to quantify the homogeneity of particle size, the histogram was fitted to normal distribution utilizing Microsoft Excel^®^. The fitted skewness parameter was 0.99, suggesting that the distribution was slightly biased to the right side. Another parameter kurtosis was 0.26, exhibiting that the particle distribution was leptokurtic (sharper peak at center than normal distribution). Thus, we could conclude that the MTX-LDH particles were well-prepared with high homogeneity.

**Figure 3:**
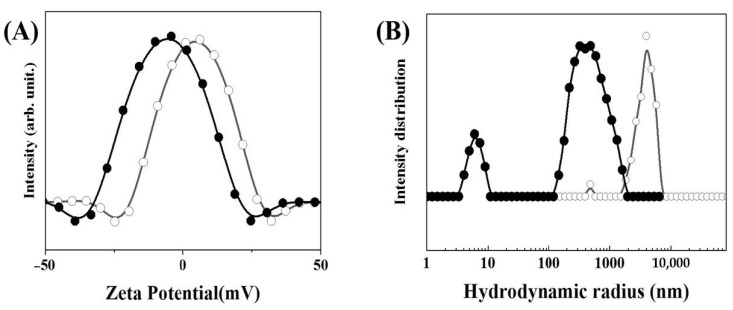
(**A**) Zeta potential distribution curves and (**B**) hydrodynamic radius distribution of MTX-LDH in neutral aqueous suspension (open circles) and simulated plasma condition (closed circles). Simulated plasma condition was prepared by dissolving fetal bovine serum in phosphate buffered saline. The concentration of sample suspension was set as ~0.1 mg/mL after optimization.

Colloidal properties of MTX-LDH, such as surface charge and hydrodynamic radius, were characterized under neutral aqueous suspension and simulated plasma condition, respectively (Figure 3). As indicated in the open circle curve in Figure 3A, the average zeta potential of MTX-LDH at neutral pH was 4.61 ± 0.97 mV, showing almost neutral surface charge. Generally, the zeta potential of LDH at neutral pH is very positive, ~+30 mV [3,55,56,57,58], due to the strong positive charge density of LDH layers. However, the zeta potential drops to the neutral value region due to the MTX moieties, which were mostly intercalated into LDH but partially covered the outer surface of LDH layers [44]. This change would result in the loss of colloidal stability due to the weak charge–charge repulsion between particles. In this regard, the hydrodynamic radius of MTX-LDH particles in neutral pH (open circles in Figure 3B) showed a larger size compared with the primary particle size observed in SEM (Figure 2). The Z-average value (4842.83 ± 197.61 nm) and the polydispersity index (PDI = (standard deviation)^2^/(average)) (1.124 ± 0.07) exhibited that MTX-LDH in neutral aqueous suspension lost its advantageous property of ~100–200 nm particle size.

However, the colloidal properties changed when MTX-LDH existed in the simulated plasma condition. Although the zeta potential (closed circles in Figure 3A) of MTX-LDH still lay in the neutral region, the hydrodynamic radius dramatically decreased with the Z-average of 287.35 ± 2.15 nm and the homogeneity increased with the PDI value of 0.38 ± 0.00 (closed circles in Figure 3B). The result encourages the idea that the MTX-LDH, once injected into the blood vessel, could take advantage of its optimum particle size ~100–200 nm under the interaction with protein. This phenomenon is attributed to the strong interaction between MTX and protein moieties, which is often exploited as the albumin-based MTX delivery system [59,60,61]. It should be noted that the MTX-LDH not only took advantage of the MTX–protein conjugation but also minimized non-specific binding of protein through the nanoparticle system.

**Figure 4:**
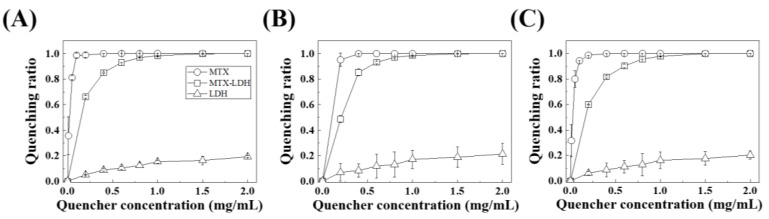
Plasma protein fluorescence quenching ratio of MTX-LDH, MTX and LDH, respectively, in (**A**) albumin, (**B**) fibrinogen, and (**C**) globulin (photoluminescence (PL) excitation wavelength: 280 nm, emission wavelength: 360 nm). The concentration of quenchers (MTX, LDH, and MTX-LDH) was varied at 0, 0.2, 0.4, 0.6, 0.8, 1.0, 1.5, and 2.0 mg/mL, while the concentration of protein (albumin, fibrinogen, and globulin) was fixed at 1.5 mg/mL.

For quantitative analyses, the fluorescence quenching effect of MTX, LDH and MTX-LDH towards representative plasma proteins—albumin, globulin, and fibrinogen—was evaluated as the quencher concentration (Figure 4). The fluorescence quenching assay has been widely applied to assess the interaction between nanoparticles and proteins [62,63,64,65] as the photoluminescence of amino acid residues such as tryptophan, tyrosine, phenylalanine etc. is sensitively affected by exogenous chemicals. For the three tested proteins, MTX showed saturated quenching at very low concentrations, 0.1, 0.4, and 0.4 mg/mL for albumin, fibrinogen, and globulin, respectively, suggesting the strong interaction between MTX and plasma proteins as previously reported [59,60,61]. On the other hand, LDH exhibited a very low quenching effect at all the tested concentrations, showing a maximum quenching ratio less than 0.2. This corresponds to our previous research that LDH could only interact with proteins through surface absorption. [66] The MTX-LDH showed an intermediate fluorescence quenching pattern, implying that it contained both properties of MTX and LDH towards proteins. The saturation quenching of MTX-LDH was found at a concentration 1.5 mg/mL, which was a sufficiently high concentration compared with MTX alone.

The fluorescence quenching behaviors of MTX, LDH, and MTX-LDH were quantitatively analyzed utilizing the Hill equation. The linear fitting results are summarized in Table 1, showing a regression factor (r^2^) higher than 0.9 for all the tested samples. It is worth taking notice of the binding constant, K_b,_ values. MTX was determined to have K_b_ values higher than 100, showing 64.2, 150, and 46.2 for albumin, fibrinogen, and globulin, respectively. On the hand, both MTX-LDH and LDH had K_b_ values less than 10. The difference in binding constant of MTX-LDH and LDH was less than double, implying that the protein binding nature of MTX-LDH is more similar to LDH than to MTX. This result suggests important features of LDH as a drug delivery carrier: (i) it can take advantage of protein corona in the plasma condition, camouflaging itself from macrophage and preventing aggregation among particles, and (ii) it affects the conformation of plasma protein at the lowest level, excluding unexpected hematotoxicity.

**Figure 5:**
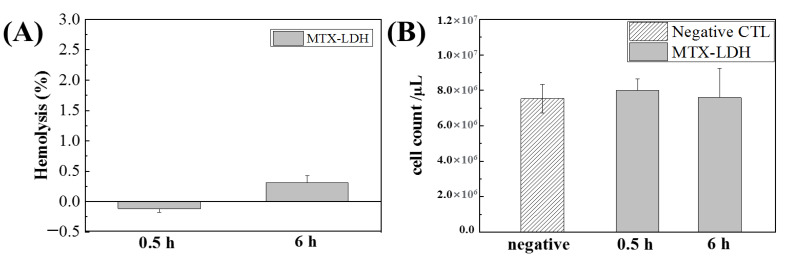
In vivo (**A**) hemolysis and (**B**) red blood cell number counting assay results of MTX-LDH after 0.5 and 6 h of injection to a Balb/c mouse. The MTX-LDH suspension (10 mg/mL) was injected (100 μL) through the tail vein, and the blood was analyzed at each time point.

The hemolytic effect of intravenously injected MTX-LDH was investigated at the administration times of 0.5 and 6 h, respectively. As shown in Figure 5A, no significant hemolysis was observed at the two time points. It was previously reported that LDH—both Mg-Al and Zn-Al composition—itself did not induce critical hemolysis at high concentrations (~10 mg/mL) under in vitro circumstances [66,67]. On the other hand, MTX has been reported to cause a significant reduction in the number of red blood cells at high dosage resulting in hemolytic anemia [65]. The injected MTX-LDH was 100 μL at 10 mg/mL concentration. Considering that, on average, a mouse has 2 mL of blood, the systemic plasma concentration of MTX-LDH was approximately 0.5 mg/mL, which was a fairly high concentration. In spite of the high dosage, the MTX-LDH did not show significant in vivo hemolysis regardless of administration time (Figure 5A).

In order to cross-confirm the low hematotoxicity of MTX-LDH, we counted the number of red blood cells at each time point (Figure 5B). The red blood cell concentration of mice without MTX-LDH treatment was approximately 7.5 × 10^6^/μL, which was similar to the previously reported cell count of Balb/c mice [68,69]. After 0.5 and 6 h of MTX-LDH administration, the cell count was determined as 7.9 × 10^6^ and 7.6 × 10^6^/μL, respectively, which were not statistically different values compared with negative control. It should be noted that MTX-LDH exhibited almost no toxicity in blood cells even at high concentration. The potential harmful effect of MTX towards blood cells would be appropriately blocked by LDH encapsulation. It was, therefore, concluded that LDH has an advantageous property as a delivery carrier by reducing the potential hematotoxicity of incorporated drug moiety.

**Figure 6:**
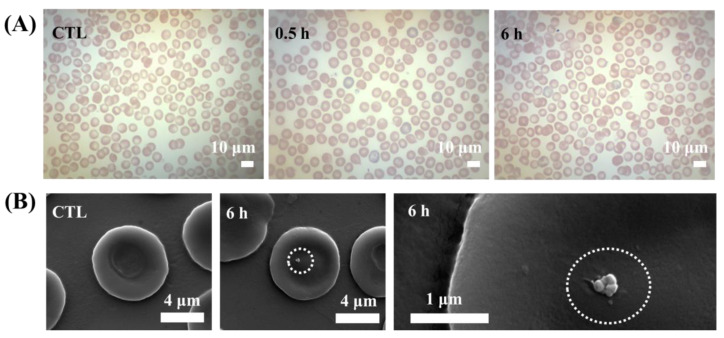
(**A**) Optical microscopy images and (**B**) SEM images of red blood cells gathered from a Balb/c mouse after injection of MTX-LDH suspension (CTL: negative control). The MTX-LDH suspension (10 mg/mL) was injected (100 μL) through the tail vein, and the blood was analyzed at each time point.

As we confirmed that MTX-LDH did not induce hemolysis nor acute reduction in the number of blood cells, the morphological change of blood cells upon MTX-LDH injection was investigated for the next step. Optical microscopy is basically utilized to evaluate disorders in red blood cells by observing morphological change. It has been frequently reported that a certain nanoparticle induces echinocyte—abnormal cell membranes characterized by many small thorny projections—or spherocyte, sphere shaped cells rather than bi-concave disc shape [70]. Titanium dioxide nanoparticles were reported to induce echinocytes at high concentration and spherocytes at low concentration [40], while nanodiamond [41] and Ag nanoparticles [43] stimulated the formation of echinocytes. Figure 6A shows the optical microscopic images of blood samples at each time point after Wright’s staining. Red blood cells without MTX-LDH treatment (Figure 6A CTL) exhibited a typical biconcave shape with a central pale area. After 0.5 and 6 h of MTX-LDH administration, we could not observe any significant morphological changes in red blood cells.

Interfacial interaction between MTX-LDH and red blood cells at 6 h of administration was visualized by SEM (Figure 6B). The magnified image showed that a cell had a lateral dimension of ~7–8 μm with biconcave disk shape. After MTX-LDH injection, we could find several particles, which were considered as MTX-LDH judging from size and shape, on the cell surface (dotted circles in Figure 6B). This kind of attachment of nanoparticles on red blood cells is often referred to as hitchhiking [71,72]. The nanoparticle-based delivery system, which takes advantage of hitchhiking, can not only boost delivery efficacy to the target organ [73] but also prolong its systemic lifetime [74,75]. Therefore, MTX-LDH can be considered fairly compatible to blood cells while its attachment on cells can further utilize the hitchhiking effect.

## 4. Conclusions

In order to investigate the colloidal properties of the LDH-based drug delivery system in blood conditions, the anticancer drug MTX was incorporated into LDH nanoparticles. According to XRD and FT-IR, it was revealed that the MTX moiety was well-encapsulated by the LDH lattice by electrostatic interaction driven layer-by-layer stacking. The SEM measurement showed that the primary particle size of MTX-LDH was ~120 nm and the size distribution was fairly narrow. MTX-LDH showed an almost neutral surface charge (~4.61 mV) and large aggregate (~4840 nm) in aqueous suspension; however, the particles were well dispersed in the physiological condition with serum, possibly due to the protein corona effect. The protein fluorescence quenching assay revealed that the behavior of MTX-LDH towards plasma protein was between MTX and LDH, while the quantitative analysis of the Hill equation showed that the binding constant of MTX-LDH to protein was closer to LDH alone. This result suggests that the LDH acted efficiently as a delivery capsule. Finally, the hemolysis effect and morphology change of red blood cells upon MTX-LDH treatment were tested in a mouse model. There were neither significant hemolysis nor morphological changes in red blood cells at 10 mg/mL of MTX-LDH injection. Furthermore, the SEM image showed that the MTX-LDH particles were attached on the red blood cells, taking advantage of the cellular hitchhiking effect. It was concluded that the LDH-based delivery system is not only compatible to blood components—blood cells and plasma protein—but also advantageous in systemic circulation.

## Figures and Tables

**Table 1:** Hill equation fitting results of the fluorescence quenching assay (PL excitation: 280 nm, emission: 360 nm). The curves in Figure 4 were fitted to the Hill plot utilizing linear regression.

Protein	Sample Name	n	K_D_	K_b_	r^2^
Albumin	MTX-LDH	3.83	2.20 × 10^−1^	4.54	0.935
	MTX	2.04	1.56 × 10^−2^	6.42 × 10^1^	0.988
	LDH	1.39	4.5 × 10^−1^	2.21	0.968
Fibrinogen	MTX-LDH	3.87	2.59 × 10^−1^	3.87	0.911
	MTX	8.50 × 10^−^^1^	6.68 × 10^−3^	1.50 × 10^2^	0.923
	LDH	1.47	4.19 × 10^−1^	2.39	0.928
Globulin	MTX-LDH	3.89	2.63 × 10^−1^	3.8	0.971
	MTX	2.45	2.17 × 10^−2^	4.62 × 10^1^	0.953
	LDH	1.39	4.45 × 10^−1^	2.25	0.939

## References

[1] Webster T.J. (2006). Nanomedicine: What’s in a definition?. Int. J. Nanomed..

[2] Deb S., Ghosh K., Shetty S.D. (2015). Nanoimaging in cardiovascular diseases: Current state of the art. Indian J. Med. Res..

[3] Kim H.-J., Lee G.J., Choi A.-J., Kim T.-H., Kim T.-i., Oh J.-M. (2018). Layered Double Hydroxide Nanomaterials Encapsulating Angelica gigas Nakai Extract for Potential Anticancer Nanomedicine. Front. Pharmacol..

[4] Ko W.C., Kim M.-S., Kwon Y.J., Jeong J., Kim W.R., Choi H., Park J.K., Jeong Y.K. (2020). Two-dimensional semiconducting covalent organic nanosheets for highly sensitive and stable NO2 sensing under humid conditions. J. Mater. Chem. A.

[5] Oh J.-M., Choi S.-J., Kim S.-T., Choy J.-H. (2006). Cellular Uptake Mechanism of an Inorganic Nanovehicle and Its Drug Conjugates:  Enhanced Efficacy Due To Clathrin-Mediated Endocytosis. Bioconjugate Chem..

[6] Angelova A., Garamus V.M., Angelov B., Tian Z., Li Y., Zou A. (2017). Advances in structural design of lipid-based nanoparticle carriers for delivery of macromolecular drugs, phytochemicals and anti-tumor agents. Adv. Colloid Interface Sci..

[7] Angelova A., Drechsler M., Garamus V.M., Angelov B. (2019). Pep-Lipid Cubosomes and Vesicles Compartmentalized by Micelles from Self-Assembly of Multiple Neuroprotective Building Blocks Including a Large Peptide Hormone PACAP-DHA. ChemNanoMat.

[8] Rakotoarisoa M., Angelov B., Espinoza S., Khakurel K., Bizien T., Angelova A. (2019). Cubic Liquid Crystalline Nanostructures Involving Catalase and Curcumin: BioSAXS Study and Catalase Peroxidatic Function after Cubosomal Nanoparticle Treatment of Differentiated SH-SY5Y Cells. Molecules.

[9] Zhang Z., Shi L., Wu C., Su Y., Qian J., Deng H., Zhu X. (2017). Construction of a Supramolecular Drug–Drug Delivery System for Non-Small-Cell Lung Cancer Therapy. ACS Appl. Mater. Interfaces.

[10] Gil E.S., Hudson S.M. (2004). Stimuli-reponsive polymers and their bioconjugates. Prog. Polym. Sci..

[11] Jung S.-Y., Gwak G.-H., Park J.K., Oh J.-M. (2020). Finely crafted quasi-core–shell gadolinium/layered double hydroxide hybrids for switching on/off bimodal CT/MRI contrasting nanodiagnostic platforms. RSC Adv..

[12] Vaccari A. (1998). Preparation and catalytic properties of cationic and anionic clays. Catal. Today.

[13] Hwang S.H., Han Y.-S., Choy J.-H. (2001). Intercalation of functional organic molecules with pharmaceutical, cosmeceutical and nutraceutical functions into layered double hydroxides and zinc basic salts. Bull. Korean Chem. Soc..

[14] Shin J., Choi C.-J., Kim T.-H., Oh J.-M. (2018). Phase Transformation from Brucite to Highly Crystalline Layered Double Hydroxide through a Combined Dissolution–Reprecipitation and Substitution Mechanism. Cryst. Growth Des..

[15] Choy J.-H., Kim Y.-K., Son Y.-H., Choy Y.B., Oh J.-M., Jung H., Hwang S.-J. (2008). Nanohybrids of edible dyes intercalated in ZnAl layered double hydroxides. J. Phys. Chem. Solids.

[16] Vasilev K., Chen H., Murray P., Mantovani D. (2015). The Potential of Nanomaterials for Drug Delivery, Cell Tracking, and Regenerative Medicine 2014. J. Nanomater..

[17] Kang H., Kim H.-J., Yang J.-H., Kim T.-H., Choi G., Paek S.-M., Choi A.-J., Choy J.-H., Oh J.-M. (2015). Intracrystalline structure and release pattern of ferulic acid intercalated into layered double hydroxide through various synthesis routes. Appl. Clay Sci..

[18] Ko S.J., Yamaguchi T., Salles F., Oh J.M. (2021). Systematic utilization of layered double hydroxide nanosheets for effective removal of methyl orange from an aqueous system by π-π stacking-induced nanoconfinement. J. Environ. Manag..

[19] Oh J.-M., Hwang S.-H., Choy J.-H. (2002). The effect of synthetic conditions on tailoring the size of hydrotalcite particles. Solid State Ion..

[20] Jung S.-Y., Kim B.-K., Hirata S., Inada M., Oh J.-M. (2020). Particle size effect of layered double hydroxide on the porosity of calcined metal oxide. Appl. Clay Sci..

[21] Choy J.-H., Kwak S.-Y., Jeong Y.-J., Park J.-S. (2000). Inorganic Layered Double Hydroxides as Nonviral Vectors. Angew. Chem. Int. Ed..

[22] Kim J.Y., Choi S.J., Oh J.M., Park T., Choy J.H. (2007). Anticancer drug-inorganic nanohybrid and its cellular interaction. J. Nanosci. Nanotechnol..

[23] Oh J.-M., Choi S.-J., Lee G.-E., Han S.-H., Choy J.-H. (2009). Inorganic Drug-Delivery Nanovehicle Conjugated with Cancer-Cell-Specific Ligand. Adv. Funct. Mater..

[24] Zuo H., Chen W., Cooper H.M., Xu Z.P. (2017). A Facile Way of Modifying Layered Double Hydroxide Nanoparticles with Targeting Ligand-Conjugated Albumin for Enhanced Delivery to Brain Tumour Cells. ACS Appl. Mater. Interfaces.

[25] Yoon Y.-s., Lee B.-I., Lee K.S., Im G.H., Byeon S.-H., Lee J.H., Lee I.S. (2009). Surface Modification of Exfoliated Layered Gadolinium Hydroxide for the Development of Multimodal Contrast Agents for MRI and Fluorescence Imaging. Adv. Funct. Mater..

[26] Choy J.-H., Jung J.-S., Oh J.-M., Park M., Jeong J., Kang Y.-K., Han O.-J. (2004). Layered double hydroxide as an efficient drug reservoir for folate derivatives. Biomaterials.

[27] Chakraborty M., Dasgupta S., Soundrapandian C., Chakraborty J., Ghosh S., Mitra M.K., Basu D. (2011). Methotrexate intercalated ZnAl-layered double hydroxide. J. Solid State Chem..

[28] Alexa I.F., Pastravanu C.G., Ignat M., Popovici E. (2013). A comparative study on long-term MTX controlled release from intercalated nanocomposites for nanomedicine applications. Colloids Surf. B Biointerfaces.

[29] Oh J.-M., Park M., Kim S.-T., Jung J.-Y., Kang Y.-G., Choy J.-H. (2006). Efficient delivery of anticancer drug MTX through MTX-LDH nanohybrid system. J. Phys. Chem. Solids.

[30] Oh J.M., Park C.B., Choy J.H. (2011). Intracellular drug delivery of layered double hydroxide nanoparticles. J. Nanosci. Nanotechnol..

[31] Kim T.-H., Lee G.J., Kang J.-H., Kim H.-J., Kim T.-i., Oh J.-M. (2014). Anticancer Drug-Incorporated Layered Double Hydroxide Nanohybrids and Their Enhanced Anticancer Therapeutic Efficacy in Combination Cancer Treatment. BioMed Res. Int..

[32] Choi S.-J., Choi G.E., Oh J.-M., Oh Y.-J., Park M.-C., Choy J.-H. (2010). Anticancer drug encapsulated in inorganic lattice can overcome drug resistance. J. Mater. Chem..

[33] Tian D.Y., Wang W.Y., Li S.P., Li X.D., Sha Z.L. (2016). A novel platform designed by Au core/inorganic shell structure conjugated onto MTX/LDH for chemo-photothermal therapy. Int. J. Pharm..

[34] Soo-Jin C., Jae-Min O., Hae-Eun C., Seung-Hee H., In-Hoo K., Jin-Ho C. (2013). In Vivo Anticancer Activity of Methotrexate-loaded Layered Double Hydroxide Nanoparticles. Curr. Pharm. Des..

[35] Choi G., Kwon O.-J., Oh Y., Yun C.-O., Choy J.-H. (2014). Inorganic Nanovehicle Targets Tumor in an Orthotopic Breast Cancer Model. Sci. Rep..

[36] Ritz S., Schöttler S., Kotman N., Baier G., Musyanovych A., Kuharev J., Landfester K., Schild H., Jahn O., Tenzer S. (2015). Protein Corona of Nanoparticles: Distinct Proteins Regulate the Cellular Uptake. Biomacromolecules.

[37] Satzer P., Svec F., Sekot G., Jungbauer A. (2016). Protein adsorption onto nanoparticles induces conformational changes: Particle size dependency, kinetics, and mechanisms. Eng. Life Sci..

[38] Dobrovolskaia M.A., Clogston J.D., Neun B.W., Hall J.B., Patri A.K., McNeil S.E. (2008). Method for analysis of nanoparticle hemolytic properties in vitro. Nano Lett..

[39] Huang H., Lai W., Cui M., Liang L., Lin Y., Fang Q., Liu Y., Xie L. (2016). An Evaluation of Blood Compatibility of Silver Nanoparticles. Sci. Rep..

[40] Wadhwa R., Aggarwal T., Thapliyal N., Kumar A., Priya, Yadav P., Kumari V., Reddy B.S.C., Chandra P., Maurya P.K. (2019). Red blood cells as an efficient in vitro model for evaluating the efficacy of metallic nanoparticles. 3 Biotech.

[41] Avsievich T., Popov A., Bykov A., Meglinski I. (2019). Mutual interaction of red blood cells influenced by nanoparticles. Sci. Rep..

[42] Yiying B., Kim K., Thien N., Kim I., Bae O.-N., Lim K.-M., Chung J.-H. (2019). Silver nanoparticles promote procoagulant activity of red blood cells: A potential risk of thrombosis in susceptible population. Part. Fiber Toxicol..

[43] Tsai L.-W., Lin Y.-C., Perevedentseva E., Lugovtsov A., Priezzhev A., Cheng C.-L. (2016). Nanodiamonds for Medical Applications: Interaction with Blood in Vitro and in Vivo. Int. J. Mol. Sci..

[44] Choi G., Kim S.Y., Oh J.-M., Choy J.-H. (2012). Drug-Ceramic 2-Dimensional Nanoassemblies for Drug Delivery System in Physiological Condition. J. Am. Ceram. Soc..

[45] Hill A.V. (1921). The Combinations of Haemoglobin with Oxygen and Carbon Monoxide, and the effects of Acid and Carbon Dioxide. Biochem. J..

[46] Hill A.V. (1913). The Combinations of Haemoglobin with Oxygen and with Carbon Monoxide. I. Biochem. J..

[47] Li L., Jiang K., Qian Y., Han H., Qiao P., Zhang H. (2020). Effect of organically intercalation modified layered double hydroxides-graphene oxide hybrids on flame retardancy of thermoplastic polyurethane nanocomposites. J. Therm. Anal. Calorim..

[48] Shabanian M., Hajibeygi M., Raeisi A., Thomas S., Daniel S. (2020). 2—FTIR characterization of layered double hydroxides and modified layered double hydroxides. Layered Double Hydroxide Polymer Nanocomposites.

[49] Cai J., Heng H.-M., Hu X.-P., Xu Q.-K., Miao F. (2016). A facile method for the preparation of novel fire-retardant layered double hydroxide and its application as nanofiller in UP. Polym. Degrad. Stab..

[50] Hashad R.A., Ishak R.A., Geneidi A.S., Mansour S. (2016). Methotrexate loading in chitosan nanoparticles at a novel pH: Response surface modeling, optimization and characterization. Int. J. Biol. Macromol..

[51] Choy J.-H., Park J.-S., Kwak S.-Y., Jeong Y.-J., Han Y.-S. (2000). Layered Double Hydroxide as Gene Reservoir. Mol. Cryst. Liquid Cryst..

[52] Lu Y., Miller J.D. (2002). Carboxyl Stretching Vibrations of Spontaneously Adsorbed and LB-Transferred Calcium Carboxylates as Determined by FTIR Internal Reflection Spectroscopy. J. Colloid Interface Sci..

[53] Oh J.-M., Choi S.-J., Lee G.-E., Kim J.-E., Choy J.-H. (2009). Inorganic Metal Hydroxide Nanoparticles for Targeted Cellular Uptake Through Clathrin-Mediated Endocytosis. Chem. Asian J..

[54] Choi S.J., Oh J.M., Choy J.H. (2008). Safety aspect of inorganic layered nanoparticles: Size-dependency in vitro and in vivo. J. Nanosci. Nanotechnol..

[55] Fang L., Li W., Chen H., Xiao F., Huang L., Holm P.E., Hansen H.C.B., Wang D. (2015). Synergistic effect of humic and fulvic acids on Ni removal by the calcined Mg/Al layered double hydroxide. RSC Adv..

[56] Kim T.-H., Hong I.T., Oh J.-M. (2018). Size- and surface charge-controlled layered double hydroxides for efficient algal flocculation. Environ. Sci. Nano.

[57] Kim H.-J., Lee S.-B., Choi A.-J., Oh J.-M. (2019). Zingiber officinale Extract (ZOE) Incorporated with Layered Double Hydroxide Hybrid through Reconstruction to Preserve Antioxidant Activity of ZOE against Ultrasound and Microwave Irradiation. Nanomaterials.

[58] Jeung D.-G., Kim H.-J., Oh J.-M. (2019). Incorporation of Glycine max Merrill Extract into Layered Double Hydroxide through Ion-Exchange and Reconstruction. Nanomaterials.

[59] Wosikowski K., Biedermann E., Rattel B., Breiter N., Jank P., Löser R., Jansen G., Peters G.J. (2003). In vitro and in vivo antitumor activity of methotrexate conjugated to human serum albumin in human cancer cells. Clin. Cancer Res. Off. J. Am. Assoc. Cancer Res..

[60] Fiehn C., Muller-Ladner U., Gay S., Krienke S., Freudenberg-Konrad S., Funk J., Ho A.D., Sinn H., Wunder A. (2004). Albumin-coupled methotrexate (MTX-HSA) is a new anti-arthritic drug which acts synergistically to MTX. Rheumatology.

[61] Li C., Wang X., Song H., Deng S., Li W., Li J., Sun J. (2020). Current multifunctional albumin-based nanoplatforms for cancer multi-mode therapy. Asian J. Pharm. Sci..

[62] Lacerda S.H., Park J.J., Meuse C., Pristinski D., Becker M.L., Karim A., Douglas J.F. (2010). Interaction of gold nanoparticles with common human blood proteins. ACS Nano.

[63] Kim H.-M., Kim K.-M., Lee K., Kim Y.S., Oh J.-M. (2012). Nano–Bio Interaction between Graphite Oxide Nanoparticles and Human Blood Components. Eur. J. Inorg. Chem..

[64] Kapur A., Aldeek F., Ji X., Safi M., Wang W., Del Cid A., Steinbock O., Mattoussi H. (2017). Self-Assembled Gold Nanoparticle–Fluorescent Protein Conjugates as Platforms for Sensing Thiolate Compounds via Modulation of Energy Transfer Quenching. Bioconjugate Chem..

[65] Li S., Aphale A.N., Macwan I.G., Patra P.K., Gonzalez W.G., Miksovska J., Leblanc R.M. (2012). Graphene Oxide as a Quencher for Fluorescent Assay of Amino Acids, Peptides, and Proteins. ACS Appl. Mater. Interfaces.

[66] Kim H.-M., Kim K.-M., Jung B.C., Kim Y.S., Choy J.-H., Oh J.-M. (2014). Hematocompatibility and Interaction of Layered Double Hydroxide Nanomaterials with Plasma Proteins. Sci. Adv. Mater..

[67] Choi S.-J., Choy J.-H. (2011). Effect of physico-chemical parameters on the toxicity of inorganic nanoparticles. J. Mater. Chem..

[68] Woolley Iii P.V., Sacher R.A., Priego V.M., Schanfield M.S., Bonnem E.M. (1983). Methotrexate-induced immune haemolytic anaemia. Br. J. Haematol..

[69] Fukuda T., Asou E., Nogi K., Goto K. (2017). Evaluation of mouse red blood cell and platelet counting with an automated hematology analyzer. J. Vet. Med. Sci..

[70] Hoffman R., Benz E.J., Silberstein L.E., Heslop H., Weitz J., Anastasi J. (2013). Hematology: Diagnosis and Treatment E-Book.

[71] Zhao Z., Ukidve A., Gao Y., Kim J., Mitragotri S. (2019). Erythrocyte leveraged chemotherapy (ELeCt): Nanoparticle assembly on erythrocyte surface to combat lung metastasis. Sci. Adv..

[72] Anselmo A.C., Kumar S., Gupta V., Pearce A.M., Ragusa A., Muzykantov V., Mitragotri S. (2015). Exploiting shape, cellular-hitchhiking and antibodies to target nanoparticles to lung endothelium: Synergy between physical, chemical and biological approaches. Biomaterials.

[73] Brenner J.S., Pan D.C., Myerson J.W., Marcos-Contreras O.A., Villa C.H., Patel P., Hekierski H., Chatterjee S., Tao J.-Q., Parhiz H. (2018). Red blood cell-hitchhiking boosts delivery of nanocarriers to chosen organs by orders of magnitude. Nat. Commun..

[74] Zelepukin I.V., Yaremenko A.V., Shipunova V.O., Babenyshev A.V., Balalaeva I.V., Nikitin P.I., Deyev S.M., Nikitin M.P. (2019). Nanoparticle-based drug delivery via RBC-hitchhiking for the inhibition of lung metastases growth. Nanoscale.

[75] Anselmo A.C., Gupta V., Zern B.J., Pan D., Zakrewsky M., Muzykantov V., Mitragotri S. (2013). Delivering Nanoparticles to Lungs while Avoiding Liver and Spleen through Adsorption on Red Blood Cells. ACS Nano.

